# Autophagy protects pancreatic beta cell mass and function in the setting of a high-fat and high-glucose diet

**DOI:** 10.1038/s41598-017-16485-0

**Published:** 2017-11-27

**Authors:** Qingfeng Sheng, Xiangwei Xiao, Krishna Prasadan, Congde Chen, Yungching Ming, Joseph Fusco, Nupur N. Gangopadhyay, David Ricks, George K. Gittes

**Affiliations:** 1 Division of Pediatric Surgery, Children’s Hospital of Pittsburgh, University of Pittsburgh School of Medicine, Pittsburgh, PA 15224 USA; 2 Department of General Surgery, Shanghai Children’s Hospital, Shanghai Jiao Tong University, No. 355, Luding Rd, Shanghai, 200062 China

**Keywords:** Obesity, Diabetes

## Abstract

Autophagy is a major regulator of pancreatic beta cell homeostasis. Altered autophagic activity has been implicated in the beta cells of patients with type 2 diabetes, and in the beta cells of obese diabetic rodents. Here, we show that autophagy was induced in beta cells by either a high-fat diet or a combined high-fat and high-glucose diet, but not by high-glucose alone. However, a high-glucose intake alone did increase beta cell mass and insulin secretion moderately. Depletion of Atg7, a necessary component of the autophagy pathway, in beta cells by pancreatic intra-ductal AAV8-shAtg7 infusion in C57BL/6 mice, resulted in decreased beta cell mass, impaired glucose tolerance, defective insulin secretion, and increased apoptosis when a combined high-fat and high-glucose diet was given, seemingly due to suppression of autophagy. Taken together, our findings suggest that the autophagy pathway may act as a protective mechanism in pancreatic beta cells during a high-calorie diet.

## Introduction

415 million people live with diabetes worldwide, and the incidence and prevalence continue to rise^1^. Type 2 diabetes (T2D), characterized by progressive beta cell failure, accounts for more than 90% of these patients. Exposure to chronic hyperglycaemia (glucotoxicity), chronic dyslipidaemia (lipotoxicity), or the combination of both (glucolipotoxicity) has been postulated to contribute to beta cell dysfunction and even beta cell loss. Moreover, a marked synergistic effect of free fatty acids and elevated glucose in inducing beta cell death has been reported in INS1 832/13 cells and in human beta cells^2–5^.

Macroautophagy (referred to hereafter as autophagy) is a fundamental eukaryotic pathway that functions to degrade and recycle aggregated proteins and damaged organelles. It plays a crucial role in the function and survival of pancreatic beta cells^6–9^. Accumulation of autophagic vacuoles and autophagosomes has been implicated in human type 2 diabetic beta cells^10^. Experimental loss of autophagy in mice has been shown to lead to reduced beta cell mass and decreased insulin secretion, indicating that autophagy is necessary for normal beta cell homeostasis^11–16^. However, over-induction of autophagy (e.g., by rapamycin treatment) impairs islet function both *in vitro* and *in vivo*^17^.

Western background diet (high-calorie) is one of the main causes of obesity and T2D. The present study was designed to investigate the effect of a high-calorie diet (high-fat diet, high-glucose water, or the combination of both) on the autophagic activity of pancreatic beta cells in mice. In addition, susceptibility of autophagy-deficient beta cells to energy-dense diet stress was also examined *in vivo*.

## Results

### Induction of autophagy in pancreatic beta cells by high-fat diet, but not by high-glucose intake

**Figure 1:**
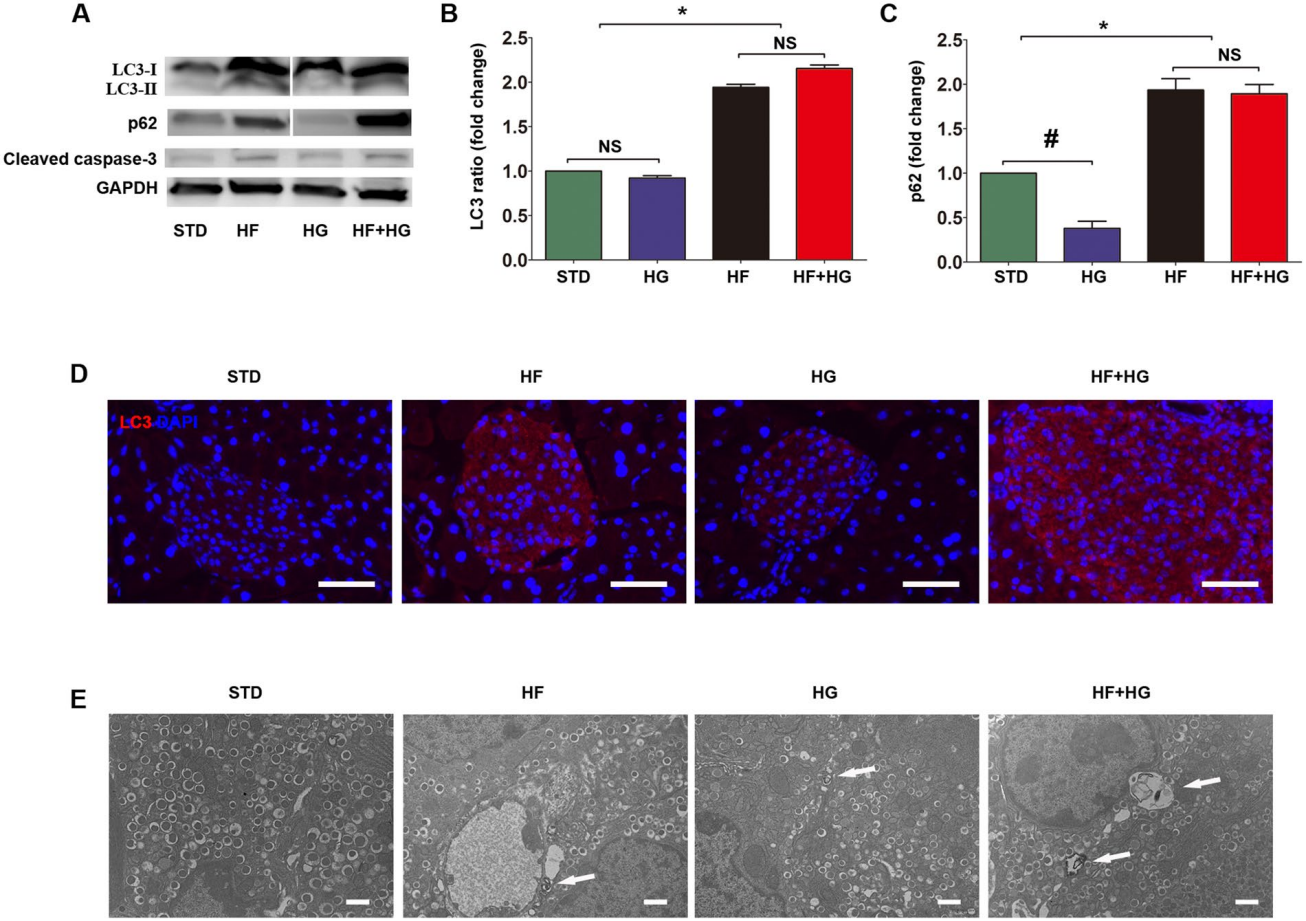
Induction of autophagy in beta cells of C57BL/6 mice receiving a high-fat diet or a combined high-fat and high-glucose feeding. Expression of LC3-I, LC3-II (**A**,**B**, see also Supplemental Fig. 7), p62 (**A**,**C**), and cleaved caspase-3 (**A**) in isolated islets from each experimental group was determined by Western blot. Cropped gels are displayed. Western blot results were analyzed by densitometry. LC3 levels were also assessed by immunostaining (**D**, scale bar, 50 μm). Beta cell ultrastructure was assessed by TEM (**E**, arrows show different sizes of autophagic vacuoles, scale bar, 1 μm). Data were expressed as mean ± SD or representative images from three to five independent experiments. *p < 0.05, HF and HF + HG versus STD or HG. ^#^p < 0.05, HG versus STD. Abbreviations: STD, standard diet; HF, high-fat diet; HG, high-glucose water; HF + HG, high-fat diet and high-glucose water; TEM, transmission electron microscopy; NS, no significant.

C57BL/6 mice were fed with standard diet (STD), high-fat diet (HF), high-glucose water (HG), or a combination of high-fat diet and high-glucose water (HF + HG) for 12 weeks starting at 6 weeks of age. Mice were injected with the lysosomal inhibitor chloroquine before being killed. Autophagy was assessed in the pancreatic islets of each group. Conversion of microtubule-associated protein 1 light chain 3-I (LC3-I) to LC3-II (increased LC3-II / LC3-I ratio, a hallmark of autophagy) was enhanced by high-fat feeding alone or by a combination of high-fat and high-glucose feeding, but not by high-glucose feeding alone (Fig. 1A,B, Supplemental Fig. 7). The presence of LC3 positive discrete fluorescent puncta reflects the presence of autophagic vacuoles. Very few fluorescent puncta were observed in islets of STD mice. However, fluorescence was enhanced in HF and HF + HG mice (Fig. 1D). We also examined autophagy by transmission electron microscopy (TEM). Autophagic vacuoles were rarely detected in beta cells of STD mice and HG mice. In contrast, beta cells of HF and HF + HG mice showed the formation of large autophagic vacuoles (Fig. 1E). p62 (also known as sequestosome 1, SQSTM1), which serves as a link between LC3 and ubiquitinated substrates, was increased in the islets of HF and HF + HG mice compared with STD mice (Fig. 1A,C). p62 and p62-bound polyubiquitinated proteins become incorporated into the autophagosome and are degraded in autolysosomes^18^. Decreased p62 levels are typically associated with autophagy activation. However, in our study, due to the use of a lysosomal inhibitor (chloroquine), decreased lysosomal proteolysis may have occurred, leading to a paradoxical observed increase in p62, as has been seen by others^13,14^. Expression of LC3-II/LC3-I ratio, p62, cleaved caspase-3, and beta cell ultrastructure from STD and HF + HG mice without chloroquine treatment similarly suggests activation of autophagy (Supplemental Fig. 1A–E). Taken together, autophagy was induced in beta cells by a high-fat diet, or a combination of high-fat and high-glucose feeding, but not by high-glucose feeding alone.

### Effects of high-fat diet and high-glucose water on beta cell mass and function

**Figure 2:**
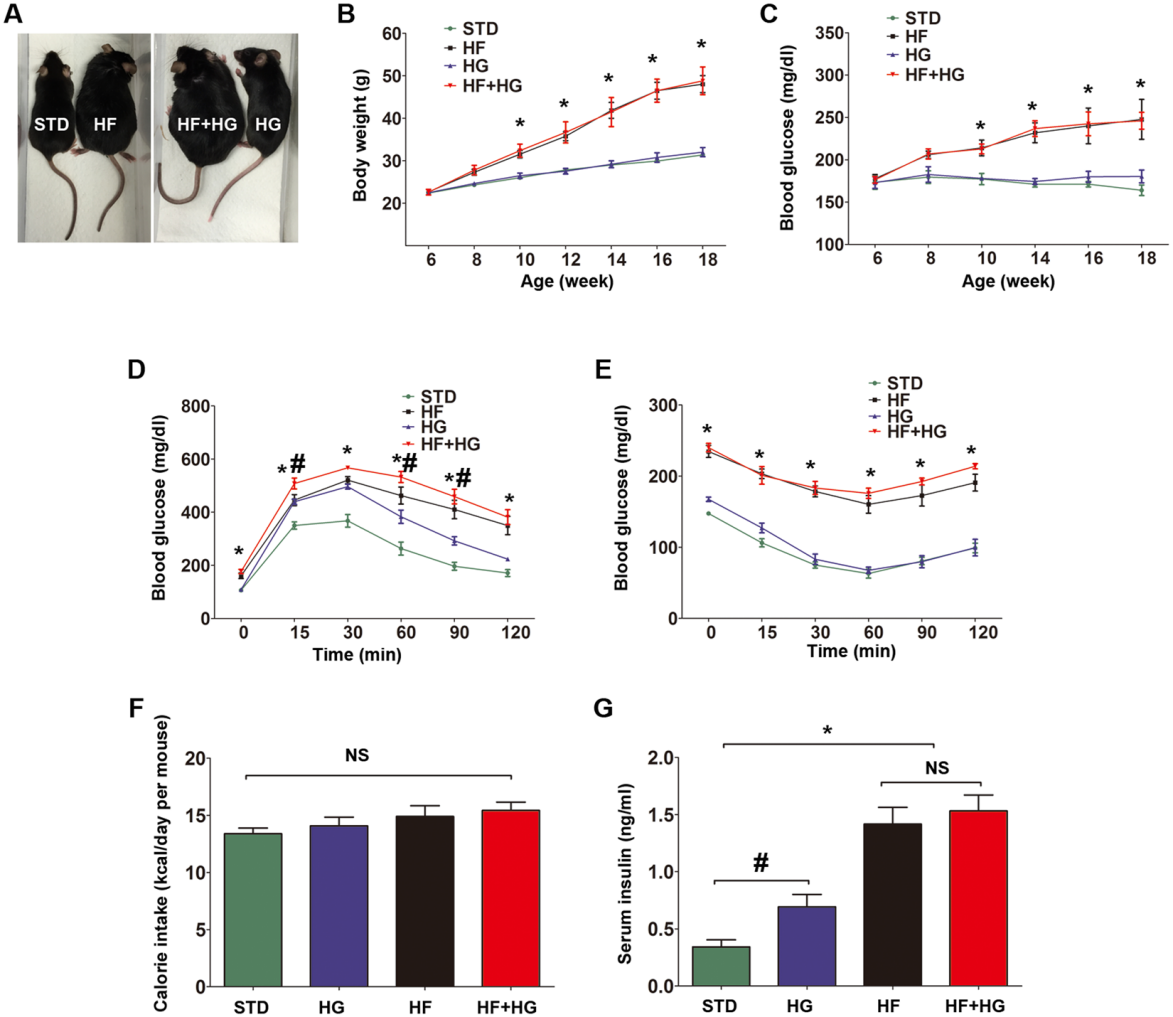
Effects of high-fat and high-glucose feeding on beta cell mass and function. Gross appearance (**A**), body weight (**B**), and random blood glucose (**C**) of each group. Intraperitoneal glucose tolerance test (**D**) and insulin tolerance test (**E**) were performed after 12 weeks of feeding. Blood glucose levels were measured at 0, 15, 30, 60, 90, and 120 min after dosing. No significant differences in calorie intake were detected among the groups (**F**). Serum insulin levels (**G**) were measured using ELISA. Data were expressed as mean ± SEM (**B**–**E**) or mean ± SD (**F**,**G**) from three to ten independent experiments. *p < 0.001, HF and HF + HG versus STD or HG. ^#^p < 0.05, HG versus STD. Abbreviations: STD, standard diet; HF, high-fat diet; HG, high-glucose water; HF + HG, high-fat diet and high-glucose water; NS, no significant; ELISA, enzyme-linked immunosorbent assay.

After 12 weeks of feeding, HF and HF + HG mice were distinguishable in appearance and growth from age-matched STD and HG mice (Fig. 2A,B). Significant increases in random blood glucose levels were detected in HF and HF + HG mice starting at 10 weeks of age (4 weeks after beginning energy-dense feeding, Fig. 2C). Calories consumed per day (kcal/day per mouse) were not different among the four groups (13.3 ± 0.5 in STD, 14.8 ± 0.9 in HF, 14.0 ± 0.7 in HG, and 15.4 ± 0.7 in HF + HG mice; p > 0.05 by ANOVA among all comparisons, Fig. 2F). Food and water intake are shown in Supplementary Fig. 2. HF and HF + HG mice displayed glucose intolerance and insulin resistance (Fig. 2D,E), and had higher concentrations of serum insulin (0.34 ± 0.1 ng/ml in STD, 1.41 ± 0.3 ng/ml in HF, 0.69 ± 0.2 ng/ml in HG, and 1.53 ± 0.3 ng/ml in HF + HG mice; HF or HF + HG vs STD, p < 0.001; Fig. 2G). HG mice also had glucose intolerance and elevated insulin levels, but not as drastic, with a p value < 0.05 (HG vs STD), instead of < 0.001. HG mice did not show insulin resistance.

**Figure 3:**
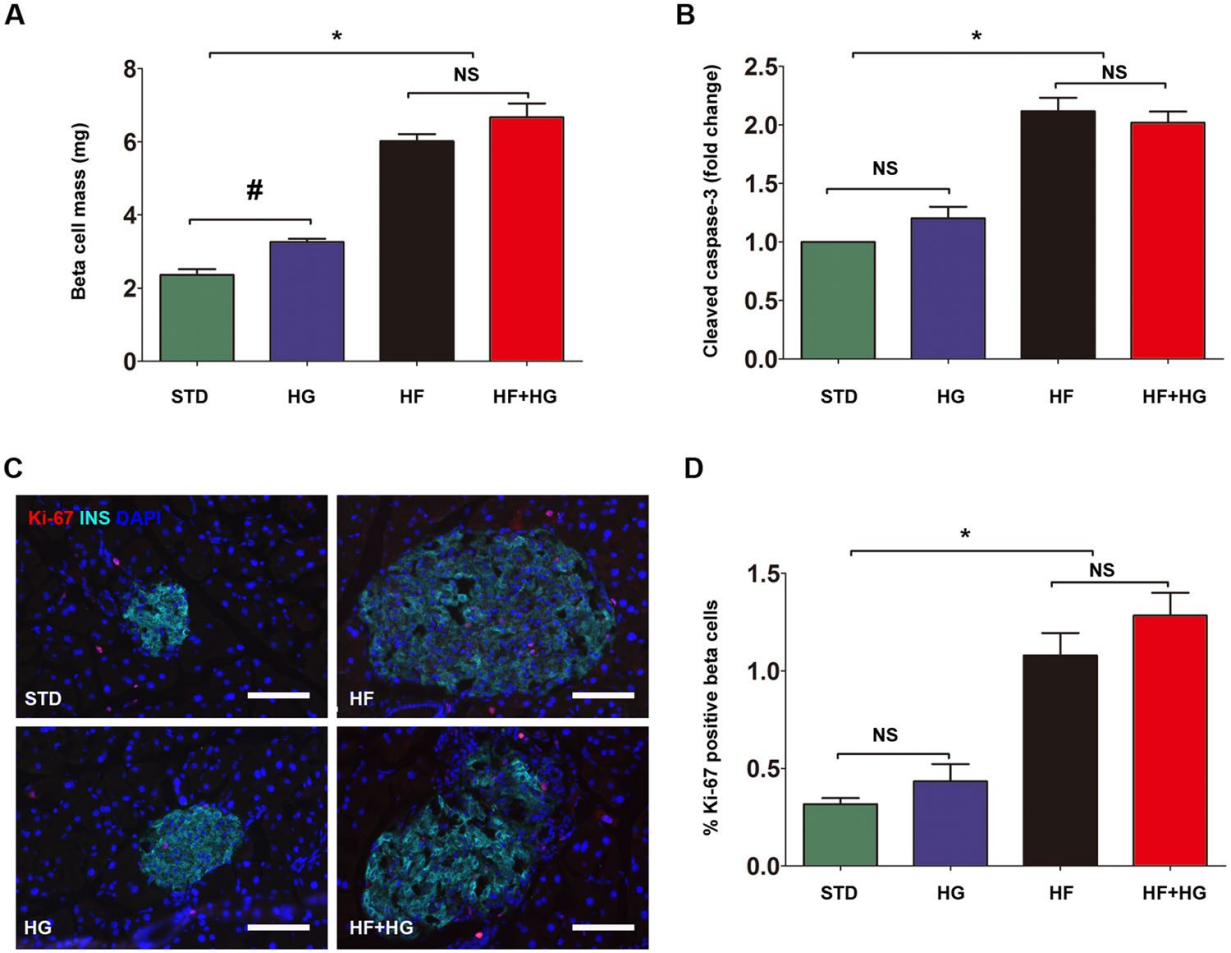
Histological analysis of pancreatic beta cells. Beta cell mass (**A**) in each group is shown. Beta cell proliferation (**C**,**D**) was analyzed by Ki-67 immunostaining. Scale bar, 50 μm. Expression of cleaved caspase-3 (**B**) in isolated islets from each group was determined by Western blot. Western blot results were analyzed by densitometry. Data were expressed as mean ± SD or representative images from three to five independent experiments. *p < 0.05, HF and HF + HG versus STD or HG. ^#^p = 0.0012, HG versus STD. Abbreviations: STD, standard diet; HF, high-fat diet; HG, high-glucose water; HF + HG, high-fat diet and high-glucose water; NS, no significant.

Next, we performed a histological analysis of pancreatic beta cells in each group. Beta cell mass was most significantly increased in HF and HF + HG mice (p < 0.001), and less so in HG mice (p = 0.0012) (2.35 ± 0.35 mg in STD, 3.25 ± 0.20 mg in HG, 6.01 ± 0.43 mg in HF, 6.56 ± 0.85 mg in HF + HG mice; Fig. 3A). Further analysis of Ki-67 immunostaining showed a significant increase in beta cell proliferation in HF and HF + HG mice (Fig. 3C,D). Additionally, cleaved caspase-3 expression, an indicator of apoptosis, was also moderately upregulated after HF or HF + HG feeding (Figs 1A and 3B). We also measured triglyceride (TG) and lipid content in liver tissue by Oil Red O (ORO) staining. Hepatic TG and lipid content in HF and HF + HG mice was higher than that of STD mice (Supplemental Fig. 3), confirming TG and lipid accumulation in liver after a high-fat diet or a combination of high-fat and high-glucose feeding.

### Morphological changes in autophagy-deficient mice after high-fat and high-glucose feeding

**Figure 4:**
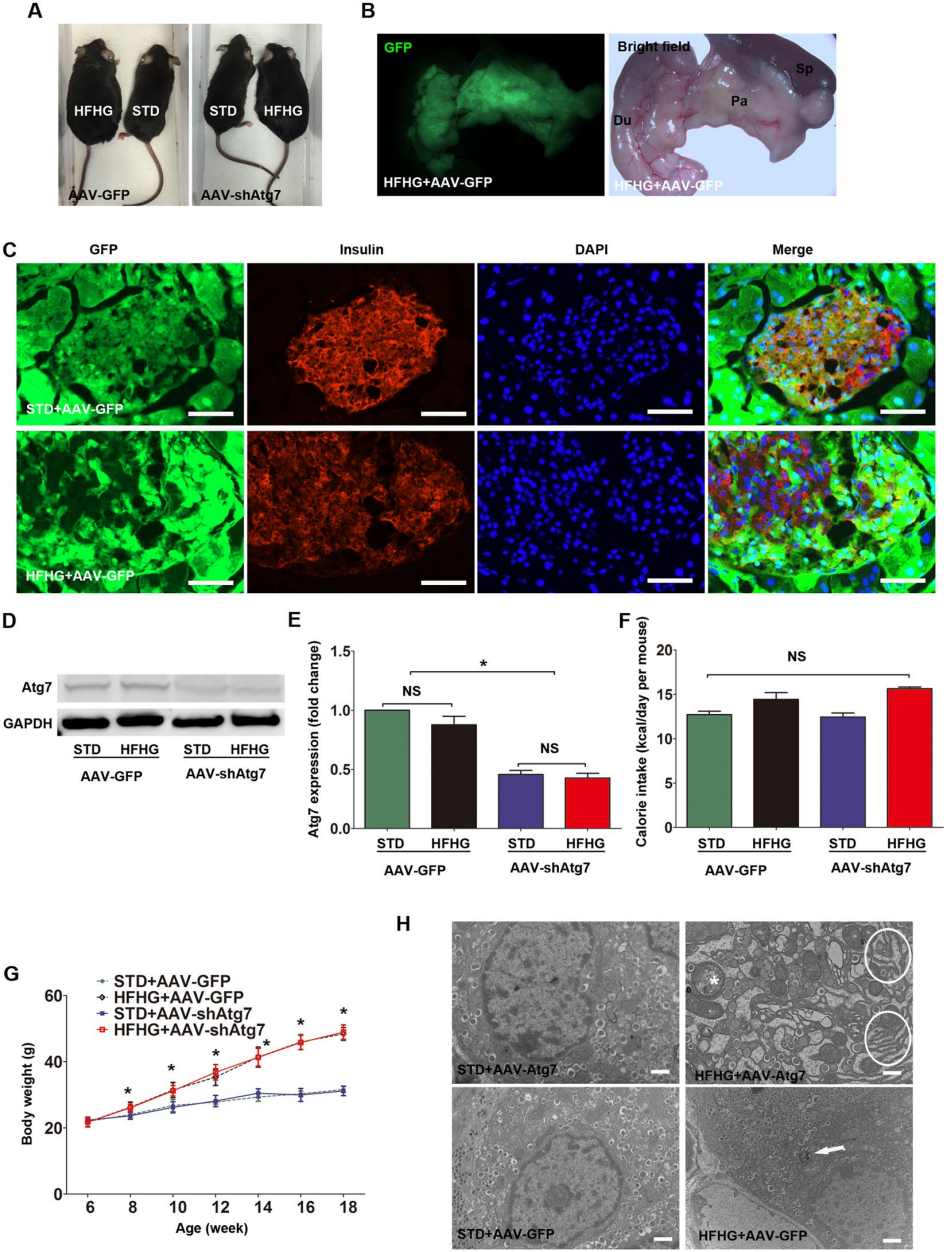
Morphological changes in autophagy-deficient mice. Gross appearance (**A**) of mice. Virus-injected pancreas was visualized under GFP fluorescence and bright field (**B**), and in pancreatic sections (**C**, scale bar, 50 μm). Expression of Atg7 (**D**,**E**) in isolated islets from each group was determined by Western blot. Western blot results were analyzed by densitometry. Calorie intake (**F**) and body weight (**G**) were also calculated. Beta cell ultrastructure was demonstrated by TEM (**H**, scale bar, 1 μm). Swollen mitochondria (asterisk), and distended endoplasmic reticulum (in white circles) were detected in HFHG + AAV-Atg7 mice. Arrows show different sizes of autophagic vacuoles. Data were expressed as mean ± SD or representative images from three to six independent experiments. *p < 0.05, HFHG + AAV-GFP and HFHG + AAV-shAtg7 versus STD + AAV-GFP or STD + AAV-shAtg7. Abbreviations: STD, standard diet; HFHG, high-fat diet and high-glucose water; TEM, transmission electron microscopy; Du, duodenum; Sp, spleen; Pa, pancreas; NS, no significant.

To explore whether impaired autophagy may affect beta cell mass and function, we generated an adeno-associated virus 8 (AAV8) bearing an shRNA against Atg7 to downregulate the expression of Atg7 (AAV-shAtg7). AAV-GFP was also generated to be used as a control. In a mouse beta cell line (βTC), Atg7 expression was reduced to 36.4% after AAV-shAtg7 transfection (Supplemental Fig. 3B). We then used our intra-ductal virus delivery system to knock down Atg7 in pancreatic islets *in vivo*. The details of the pancreatic intra-ductal infusion technique have been reported by our group recently^19,20^. Standard diet fed or high-fat + high-glucose fed C57BL/6 mice received either AAV-shAtg7 (STD + AAV-shAtg7, HFHG + AAV-shAtg7), or an identical titer of AAV-GFP as a control (STD + AAV-GFP, HFHG + AAV-GFP). GFP^+^ pancreas and islets were visualized grossly, and in pancreatic sections (Fig. 4B,C). Atg7 expression was quantified in isolated islets from AAV-shAtg7-infused mice, showing a significant decrease compared with islets from AAV-GFP-infused mice (0.87 ± 0.12 in HFHG + AAV-GFP, 0.42 ± 0.06 in HFHG + AAV-shAtg7; p = 0.0054; Fig. 4D,E). HFHG + AAV-shAtg7 and STD + AAV-shAtg7 mice were indistinguishable in appearance and growth from age-matched HFHG + AAV-GFP and STD + AAV-GFP mice, respectively (Fig. 4A,G). Food intake, water intake, and calories consumed per day were not different between HFHG + AAV-shAtg7 and HFHG + AAV-GFP mice (Fig. 4F, Supplemental Fig. 4C,D). TEM showed severely swollen mitochondria and distension of the endoplasmic reticulum in beta cells of HFHG + AAV-shAtg7 mice (Fig. 4H). No significant differences in morphological changes were observed between STD + AAV-GFP and STD + AAV-shAtg7 mice. Additionally, our data showed AAV infusion induced no or mild local inflammation in the pancreas using a pan-leukocyte marker CD45 immunostaining (Supplemental Fig. 5).

**Figure 5:**
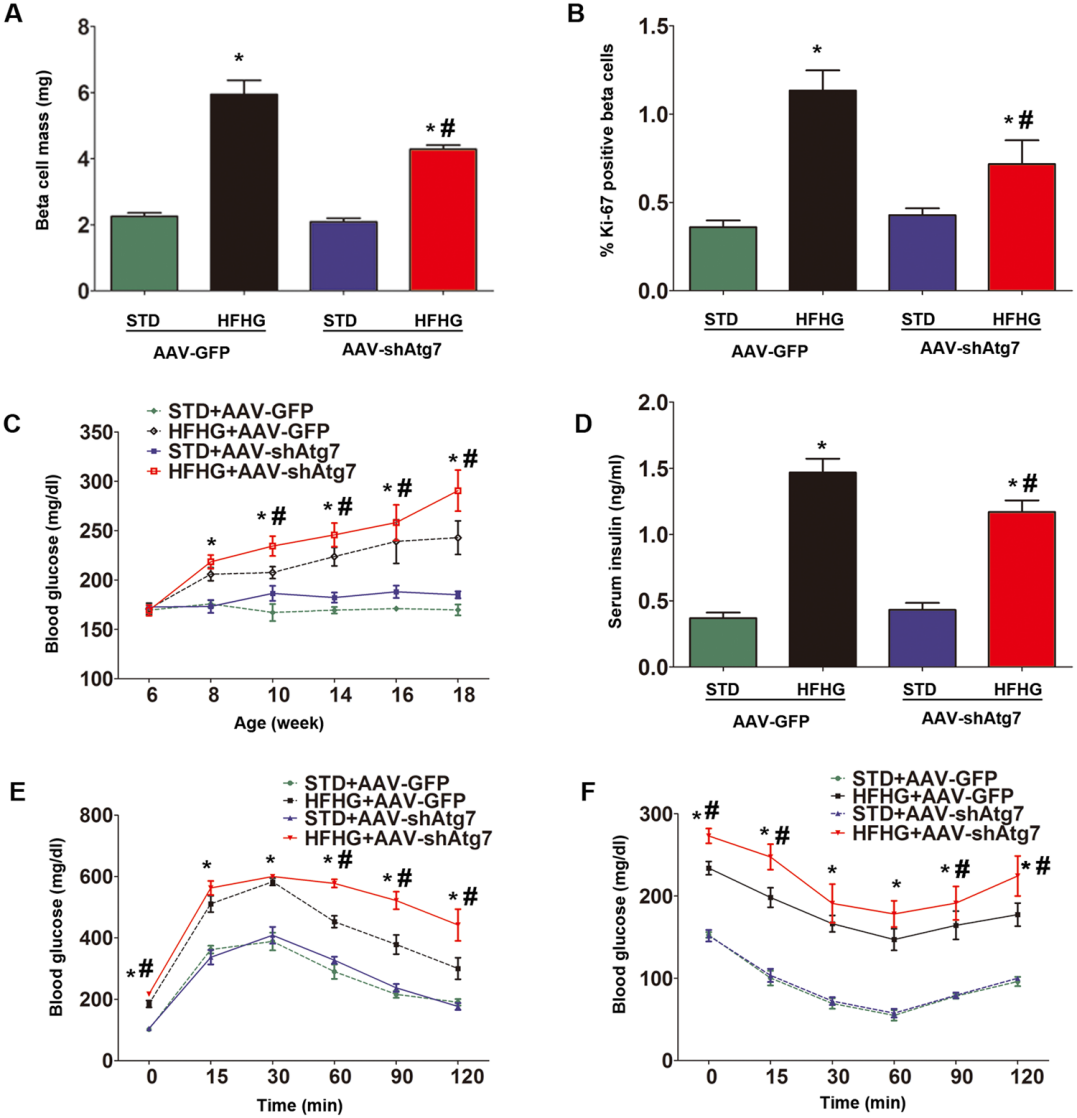
Deterioration of beta cell mass and function in autophagy-deficient mice after high-fat and high-glucose feeding. Beta cell mass (**A**), beta cell proliferation (**B**), and random blood glucose (**C**) were measured in each group. Serum insulin levels (**D**) were measured using ELISA. Intraperitoneal glucose tolerance test (**E**) and insulin tolerance test (**F**) were performed after 12 weeks of feeding. Blood glucose levels were examined at 0, 15, 30, 60, 90, and 120 min after dosing. Data were expressed as mean ± SD from three to six independent experiments. *p < 0.05, HFHG + AAV-GFP and HFHG + AAV-shAtg7 versus STD + AAV-GFP or STD + AAV-shAtg7. ^#^p < 0.05, HFHG + AAV-GFP versus HFHG + AAV-shAtg7. Abbreviations: STD, standard diet; HFHG, high-fat diet and high-glucose water; ELISA, enzyme-linked immunosorbent assay.

### Susceptibility of autophagy-deficient mice to beta cell injury from high-fat and high-glucose feeding

Next, we investigated whether autophagy deficiency may affect beta cell mass and function after high-fat and high-glucose feeding. Twelve weeks after AAV infusion and HFHG feeding, beta cell mass in mice that received AAV-shAtg7 was significantly reduced when compared with mice that received AAV-GFP (5.94 ± 0.95 mg in HFHG + AAV-GFP, 4.28 ± 0.26 mg in HFHG + AAV-shAtg7; p = 0.0058; Fig. 5A), indicating some failure of beta cell compensatory proliferation. The impaired capacity of beta cell proliferation was further confirmed by analysis of Ki-67 immunostaining (Fig. 5B). Moreover, HFHG + AAV-shAtg7 mice developed higher random blood glucose concentrations (Fig. 5C) and lower serum insulin levels (1.46 ± 0.2 ng/ml in HFHG + AAV-GFP, 1.17 ± 0.2 ng/ml in HFHG + AAV-shAtg7 mice; p < 0.05; Fig. 5D). IPGTT and intraperitoneal insulin tolerance test (IPITT) of HFHG + AAV-shAtg7 mice showed severe glucose intolerance and insulin resistance compared with HFHG + AAV-GFP mice (Fig. 5E,F). No significant differences in beta cell mass and function were detected between STD + AAV-shAtg7 and STD + AAV-GFP mice. TG and lipid accumulation in the liver were observed similarly in HFHG + AAV-shAtg7 and HFHG + AAV-GFP mice (Supplemental Fig. 6).

**Figure 6:**
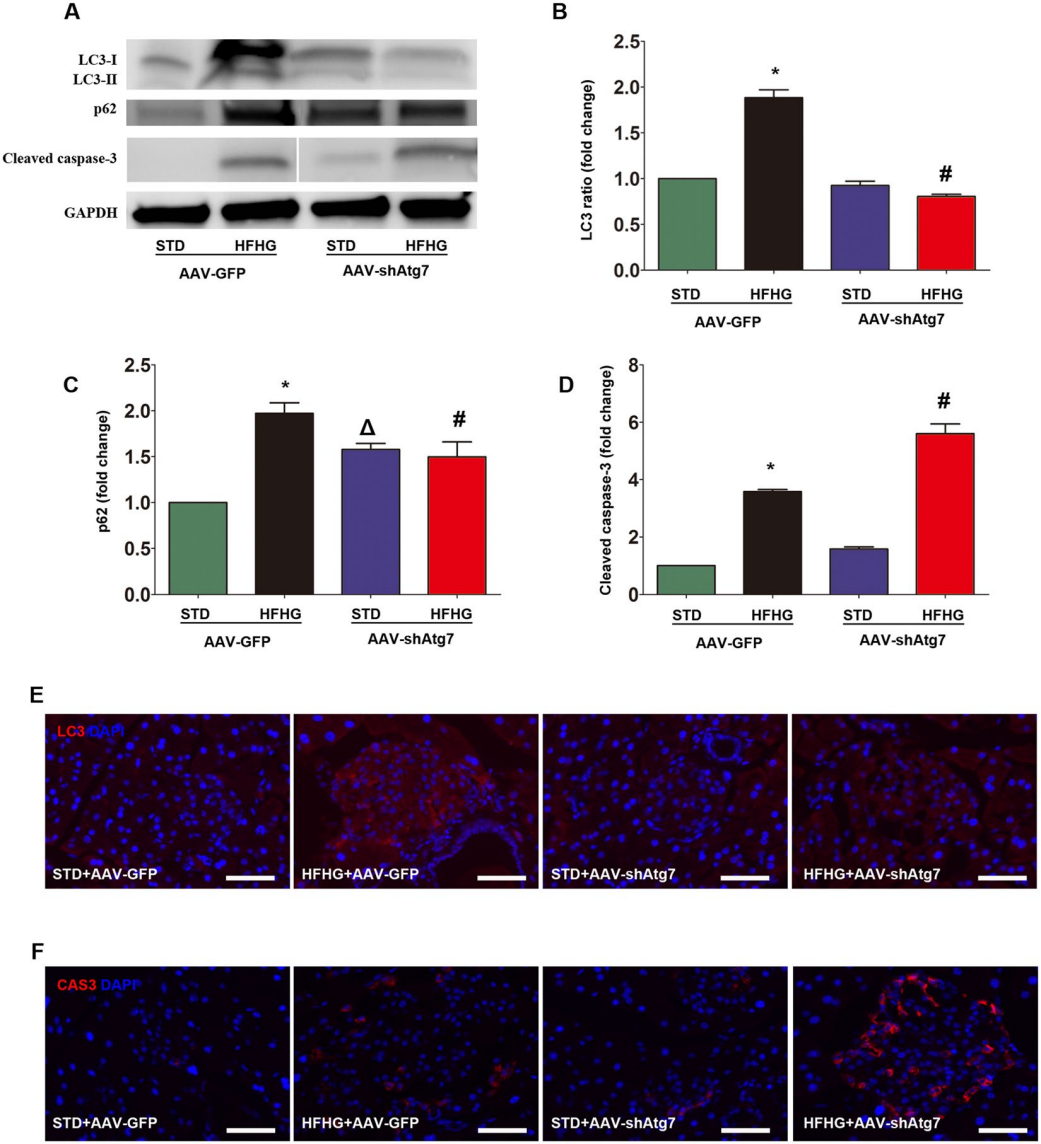
Apoptosis increased in autophagy-deficient mice after high-fat and high-glucose feeding. Expression of LC3-I, LC3-II (**A**,**B**), p62 (**A**,**C**), and cleaved caspase-3 (**A**,**D**, see also Supplemental Fig. 7) in isolated islets from each group was determined by Western blot. Cropped gels are displayed. Western blot results were analyzed by densitometry. LC3 levels and cleaved caspase-3 expression were also detected by immunostaining (**E** and **F**, scale bar, 50 μm). Data were expressed as mean ± SD or representative images from three to five independent experiments. *p < 0.05, HFHG + AAV-GFP versus either STD + AAV-GFP, or STD + AAV-shAtg7. ^#^p < 0.05, HFHG + AAV-GFP versus HFHG + AAV-shAtg7. Δ p < 0.05, STD + AAV-GFP versus STD + AAV-shAtg7. Abbreviations: STD, standard diet; HFHG, high-fat diet and high-glucose water.

### Impaired autophagy increases apoptosis of pancreatic beta cells after high-fat and high-glucose feeding

Impaired autophagy in mice (chloroquine treatment before being killed) that received an AAV-shAtg7 infusion (STD + AAV-shAtg7, HFHG + AAV-shAtg7) was confirmed by analysis of the conversion of LC3-I to LC3-II, and by LC3 immunostaining (Fig. 6A,B,E). The expression of another autophagy marker, p62, was reduced in islets of HFHG + AAV-shAtg7 mice compared with HFHG + AAV-GFP mice (Fig. 6A,C). Accumulation of p62 was observed in STD + AAV-shAtg7 mice. Apoptosis is the main cell death mechanism in beta cells in human type 2 diabetes. Thus, we investigated the expression of cleaved caspase-3, a hallmark of apoptosis, by Western blot and immunostaining. In agreement with previous reports^11,12,15^, cleaved caspase-3 was significantly elevated in islets of HFHG + AAV-shAtg7 mice (Fig. 6A,D,F, Supplemental Fig. 7), indicating that decreased autophagy was associated with increased apoptosis in pancreatic beta cells.

## Discussion

Autophagy is crucially involved in the maintenance of energy homeostasis under nutrient-limited situations (e.g., starvation). Interestingly, dysregulation of autophagy contributes to many metabolic diseases, including obesity, diabetes, and atherosclerosis^6,9,21,22^. Our study showed that autophagic vacuoles were upregulated in pancreatic beta cells after either a high-fat feeding alone, or in combination with high-glucose intake, consistent with results in islets from high fat-fed wild-type mice, db/db mice, patients with type 2 diabetes, and *in vitro* models of beta cell glucolipotoxicity^4,11,15^. No significant effects on body weight and random blood glucose were observed after 12 weeks of high-glucose drinking (HG mice). However, high-glucose intake alone did lead to increased beta cell mass and insulin secretion, indicating the occurrence of a beta cell adaptive response. Hyperglycaemia and dyslipidaemia are common features of T2D. The esterification pathway of fatty acids is preferentially activated in the context of high glucose, resulting in a cytosolic accumulation of lipid-derived molecules such as ceramide, phospholipids, and triglycerides^5^. In addition, high glucose activates the expression of genes involved in lipogenesis and cholesterol metabolism^5,23^.

Our second finding was that impaired autophagy increased susceptibility of beta cells to injury from a high-calorie intake (high-fat and high-glucose feeding, a common condition in developed countries), leading to dysfunction and degeneration of beta cells. This finding was in line with recent studies reported by Ebato *et al*.^11^ and Quan *et al*.^13^. The expression of Atg7, an E1-like key enzyme, was downregulated by pancreatic intra-ductal virus infusion in our study. Contrary to the results of beta cell-specific Atg7-knockout (Atg7^Δβcell^) mice, standard diet-fed AAV-shAtg7 mice (STD + AAV-shAtg7) did not demonstrate hyperglycaemia, glucose intolerance, nor insulin resistance. This difference may be explained by the possibility that enough autophagy could still be maintained (despite the shRNA knock-down) to adapt to minor physiologic stresses.

Cell death can be classified into four modes by morphological changes: apoptosis, autophagy, necrosis, and mitotic catastrophe^24^. Apoptosis has long been considered a major beta cell death mechanism in type 2 diabetes. Our results here and those of others^11–13^ suggest that autophagy-deficient mice experience increased beta cell apoptosis. Interestingly, Komiya *et al*.^25^ showed that short-term (within 6 hours) exposure to palmitate could activate autophagy through the PKR-JNK1 pathway. It is conceivable that a reciprocal relationship exists between autophagy and apoptosis when beta cells are subjected to oxidative stress and endoplasmic reticulum stress (i.e., free fatty acids, high glucose).

The main limitation of our study was that the Atg7 knockdown was not beta cell specific. Intra-ductal virus infusion might affect the function of pancreatic exocrine cells (acinar cells) and other endocrine cells (e.g., alpha cells). Antonucci *et al*.^26^ showed that Pdx1-Cre; Atg7^f/f^ (Atg7^Δpan^) mice, in which Atg7 is deleted from the entire pancreas, exhibited pancreatic degeneration, inflammation and fibrosis. Thus, the unexpected effects of AAV-shAtg7 on other pancreatic cell types need further elucidation. The roles of mTOR signaling and mitochondrial oxidative or ER stress in autophagy-deficient pancreatic beta cells also need to be investigated.

In summary, the data from our study indicate that autophagy deficiency led to deteriorated beta cell mass and function after high-fat and high-glucose feeding. Our findings provide further evidence that autophagy is a key regulator of pancreatic beta cell homeostasis in response to energy-dense diets.

## Materials and Methods

### Cell cultures, reagents, and antibodies

The mouse beta cell line (βTC) was cultured in Dulbecco’s modified Eagle’s medium (DMEM) containing 10% fetal bovine serum (Gibco, Carlsbad, CA, USA) and 25 mM glucose. Chemicals, including glucose, chloroquine, and 4′,6-diamidino-2-phenylindole (DAPI), were purchased from Sigma-Aldrich (St. Louis, MO, USA). Primary antibodies, including anti-Atg7, p62, LC3A/B, Ki-67, cleaved caspase-3, CD45, and glyceraldehyde-3-phosphate dehydrogenase (GAPDH) were obtained from Cell Signaling Technology (Beverly, MA, USA).

### Mouse manipulation

All mouse experiments were approved by the Animal Research and Care Committee at the Children’s Hospital of Pittsburgh and the University of Pittsburgh Institutional Animal Care and Use Committee. It is confirmed that all methods were performed in accordance with the relevant guidelines and regulations. C57BL/6 mice were purchased from Jackson Laboratory (Bar Harbor, ME, USA). Male C57BL/6 mice 6 weeks of age were caged with free access to fresh water or high-glucose water (10% glucose), fed a standard diet (LabDiet, St. Louis, MO, USA) or high-fat diet (Research Diets, New Brunswick, NJ, USA). The composition of both diets is shown in Supplemental Table 1. Pancreatic intra-ductal virus infusion was performed as we reported previously^19,20,27,28^. Briefly, 6- week-age mice were anesthetized with isoflurane by inhalation first. The duodenum was rotated and stretched to expose the biliary-pancreatic duct. A microclamp was placed on the bile duct to prevent perfusion of the liver. A small hole was made opposite to the sphincter of Oddi by a 30-gauge needle. Then a 31-gauge blunted catheter (World Precision Instruments, Sarasota, FL, USA) was placed into the common bile duct carefully. The back end of the catheter was connected with a microinfusion apparatus. 150 μl AAV8 virus was infused at the rate of 10 μl/min. After infusion, removed the microclamp, withdrew the catheter, and closed the abdomen incision. Mice were injected with the lysosomal inhibitor chloroquine (20 mg/kg per day) for 5 days before sacrifice.

### Blood glucose measure, glucose tolerance test, insulin tolerance test

Blood glucose concentration was measured using ACCU-CHEK glucose meter (Roche, Basel, Switzerland) as described previously^13,14,19^. For intraperitoneal glucose tolerance test (IPGTT), 16-hour-fasted mice were injected with 2 g/kg glucose. For intraperitoneal insulin tolerance test (IPITT), 6-hour-fasted mice were given 0.75 mU/g insulin. Then, blood glucose levels were detected at 0, 15, 30, 60, 90, and 120 min after dosing.

### Serum insulin measure

Blood was collected from the tail vein, clotted for 30 min before centrifugation at 3000 rpm for 10 min. Insulin concentration in the serum was measured using mouse ultrasensitive insulin enzyme-linked immunosorbent assay (ELISA) kit (Alpco, Salem, NH, USA).

### Beta cell mass and beta cell proliferation

Beta cell mass was determined as described before^19^. Briefly, cryosections at 100 μm interval of the whole pancreas were stained with insulin and DAPI. Then the cross sectional area of beta cells and cross sectional area of total tissue were analyzed by ImageJ software (NIH Image, National Institute of Health, Bethesda, MD, USA). The beta cell mass per pancreas was calculated by the value of relative cross sectional area of beta cells and the weight of pancreas. Quantification of beta cell proliferation was determined by Ki-67 positive beta cells. At least 2000 beta cells were counted for each mouse.

### Virus production

The plasmid DNA were obtained from ViGene Biosciences (Rockville, MD, USA). Endotoxin-free plasmid DNA was generated and purified by NucleoBond Xtra Midi Plus EF kit (Macherey-Nagel, Dueren, Germany). βTC cells were transfected with plasmid DNA containing Atg7 shRNA, or negative control using Lipofectamine 3000 (ThermoFisher, Waltham, MA, USA). The efficacy of knockdown was assessed. Adeno-associated virus (AAV) serotype 8 vectors were generated by polyethylenimine (PEI) transfection of HEK-293 cells as previously reported^19,20,27,28^. AAV was purified using discontinuous iodixanol gradients. Purified AAV vectors were filtered and stored at −80°.

### Transmission electron microscopy (TEM)

Ultrastructural changes of pancreatic beta cells were examined using TEM (JEM-1400, JEOL, Tokyo, Japan). Briefly, the pancreas samples were fixed in 2.5% glutaraldehye in 0.1 M phosphate buffer (pH 7.2). Tissues were then post-fixed in 1% osmium tetroxide containing 1% potassium ferrycyanide. After dehydration, samples were embedded in pure epon for cutting of ultrathin sections. Ultrathin sections were stained with uranyl acetate and lead citrate before imaging.

### Immunostaining, islet isolation, western blot

Pancreas and liver were harvested and fixed in 4% paraformaldehyde overnight, followed by 30% sucrose incubation. Then, samples were OCT embedded, frozen, and microtome sectioned at 6 μm. The procedures of immunostaining were performed as described before^19,29,30^. Secondary antibodies for fluorescent staining were Cy2-, Cy3-, or Cy5-conjugated anti-guinea pig, or anti-rabbit (Jackson ImmunoResearch, West Grove, PA, USA). Nuclei were stained with DAPI. Mouse islets were isolated by thermolysin and liberase (Roche Diagnostics, Basel, Switzerland) perfusion into the common bile duct, pancreas digestion, islet purification, and islet picking. Total proteins were extracted from isolated islets, and quantified by bicinchoninic acid method (BCA kit, Thermo Scientific, Waltham, MA, USA). SDS-PAGE was also performed as described previously^29^. Proteins were transferred to PVDF membrane, and blotted with primary antibodies, and then identified by reacting the membrane with HRP-conjugated anti-rabbit secondary antibody (Jackson Labs, Bar Harbor, ME, USA), followed by enhanced chemiluminescence (ECL).

### Oil Red O staining

For detection of triglyceride and lipid droplet, cryosections of liver were stained with a working solution comprising of 0.5 g Oil Red O (Sigma, St. Louis, MO, USA) in 100 ml isopropanol with water (3:2) for 20 min. After washing with water, the slides were stained with hematoxylin before imaging.

### Statistical analysis

Statistical analysis was performed using the SPSS 17.0 software package. Results were expressed as mean ± SD or mean ± SEM. The differences among four groups were detected by one-way analysis of variance (ANOVA) and Students-Newman-Keuls post hoc test. Difference was considered to be significant when p < 0.05.

## Electronic supplementary material

Supplementary Information
