# Supplementary material for: Autophagy protects pancreatic beta cell mass and function in the setting of a high-fat and high-glucose diet
Source: Sci Rep. 2017 Nov 27;7:16348. doi: 10.1038/s41598-017-16485-0 (PMC5703965; doi:10.1038/s41598-017-16485-0)

Autophagy protects pancreatic beta cell mass and function in the setting of a high-fat and high-glucose diet

Qingfeng Sheng, Xiangwei Xiao, Krishna Prasadan, Congde Chen, Yungching Ming, Joseph Fusco, Nupur N. Gangopadhyay, David Ricks, George K. Gittes

Supplemental Table 1 Composition of the regular diet and high-fat diet.

	Regular diet	High-fat diet
Protein (%)	22.5	26.2
Fat (%)	5.4	34.9
Carbohydrates (%)	52.0	26.3
Energy (kcal/g)	3.20	5.24

Supplemental Figure legends

Supplemental Figure 1. Induction of autophagy in beta cells of C57BL/6 mice (without chloroquine treatment) receiving a combined high-fat and high-glucose feeding. Expression of LC3-I, LC3-II (A,B), p62 (A,C), and cleaved caspase-3 (A,D) in isolated islets from each experimental group was determined by Western blot. Western blot results were analyzed by densitometry. Beta cell ultrastructure was assessed by TEM (E, arrow shows autophagic vacuole, scale bar, 1 μm). Data were expressed as mean ± SD or representative images from three to five independent experiments. * $p < 0.05$, HF+HG versus STD. Abbreviations: STD, standard diet; HF+HG, high-fat diet and high-glucose water; TEM, transmission electron microscopy.

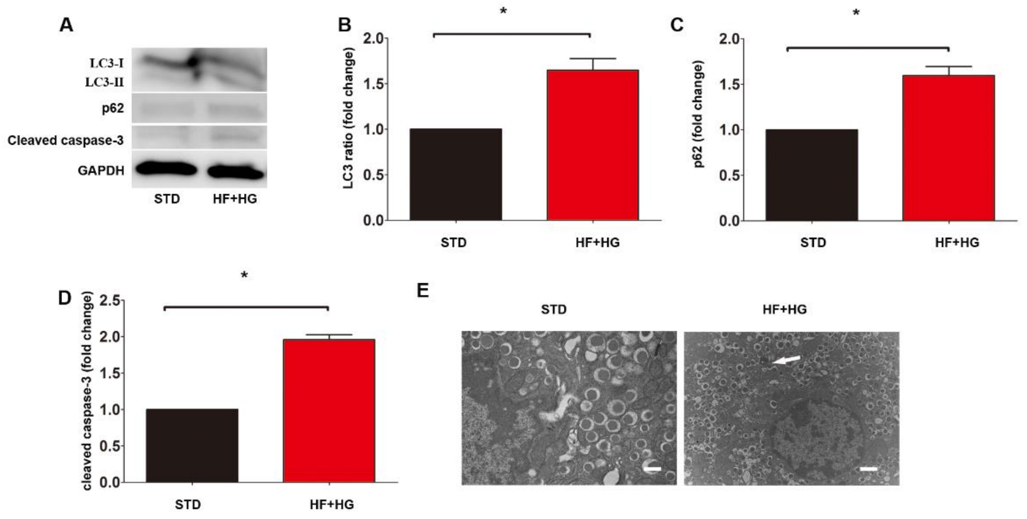

Supplemental Figure 2. Food intake (A) and water intake (B) of each group are shown. * $p < 0.05$, STD versus HF, HG or HF+HG. # $p < 0.05$, HG versus STD, HF, or HF+HG. Data were expressed as mean ± SD from six independent experiments. Abbreviations: STD, standard diet; HF, high-fat diet; HG, high-glucose water; HF+HG, high-fat diet and high-glucose water; NS, no significant.

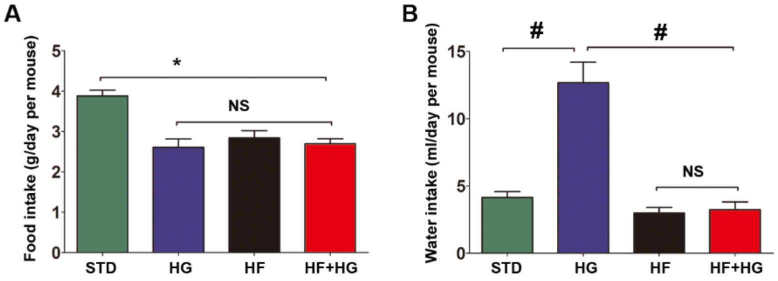

Supplemental Figure 3. Triglyceride and lipid droplet accumulation in the liver of HF and HF+HG mice. Representative images of Oil Red O staining are shown. Scale bar, 50 μm. Abbreviations: STD, standard diet; HF, high-fat diet; HG, high-glucose water; HF+HG, high-fat diet and high-glucose water.

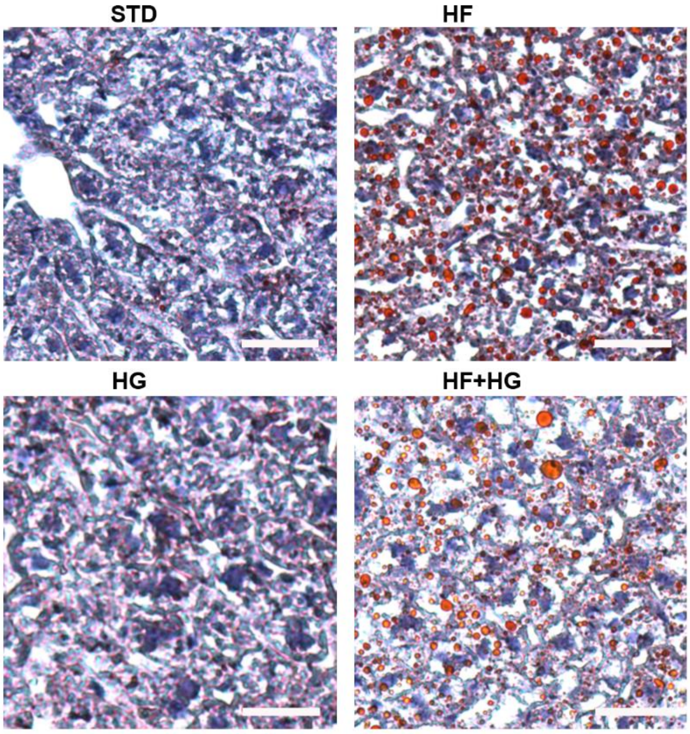

Supplemental Figure 4. βTC cells were cultured and transfected with different Atg7 shRNA (1#, 2#, 3#, 4#), or control vector. Proteins were extracted from cells after 48 h incubation. Expression of Atg7 was determined by Western blot (A, * $p < 0.05$, Atg7 shRNA versus control or GFP vector). The #2 Atg7 shRNA was chosen to generate AAV. βTC cells were cultured in 6-well plates and transfected with different concentrations of AAV-shAtg7. Proteins were extracted from cells 72 h after transfection. Expression of Atg7 was analyzed by Western blot (B, * $p < 0.05$, virus versus control). Western blot results were analyzed by densitometry. Food intake (C) and water intake (D) of each group were shown. Data were expressed as mean ± SD or representative images from three to six independent experiments. * $p < 0.05$, HFHG + AAV-GFP and HFHG + AAV-shAtg7 versus STD + AAV-GFP or STD + AAV-shAtg7. Abbreviations: STD, standard diet; HF+HG, high-fat diet and high-glucose water; NS, no significant.

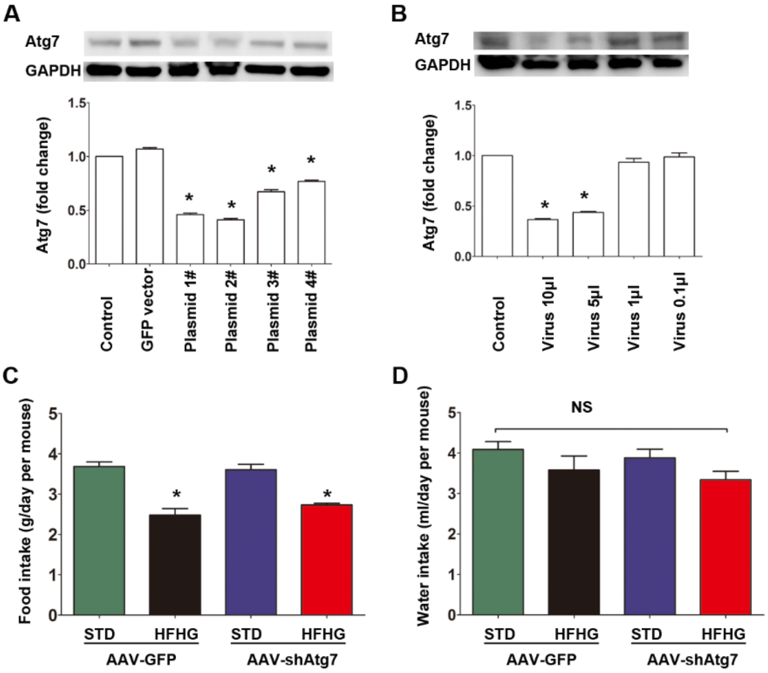

Supplemental Figure 5. Intra-ductal AAV infusion induced no or slight inflammation in the pancreas. Inflammation was detected by immunostaining for CD45 (a panleukocyte marker). Representative images are shown. Scale bar, 50 µm. Abbreviations: STD, standard diet; HFHG, high-fat diet and high-glucose water.

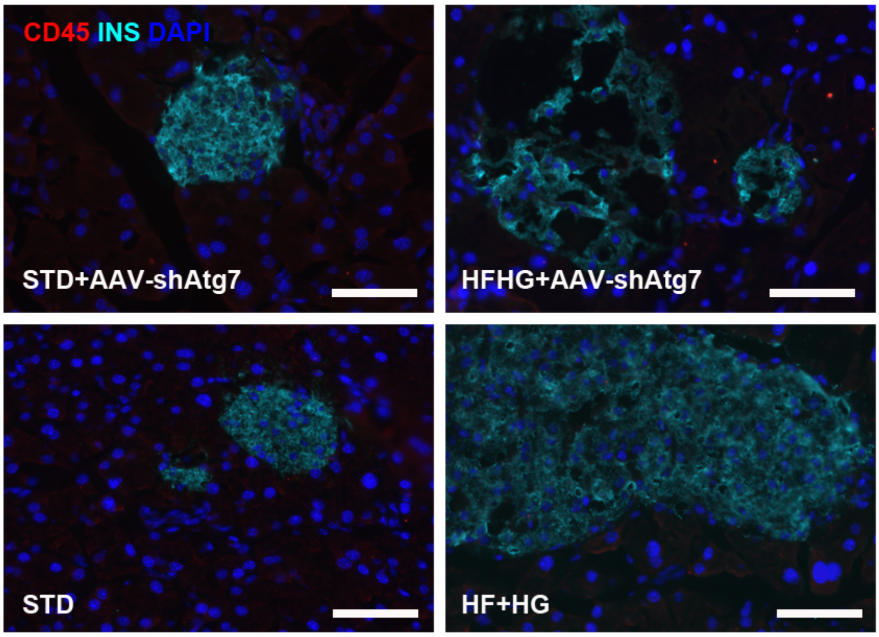

Supplemental Figure 6. Triglyceride and lipid droplet accumulation in livers of HFHG + AAV-GFP and HFHG + AAV-shAtg7 mice. Representative images of Oil Red O staining are shown. Scale bar, 50 µm. Abbreviations: STD, standard diet; HFHG, high-fat diet and high-glucose water.

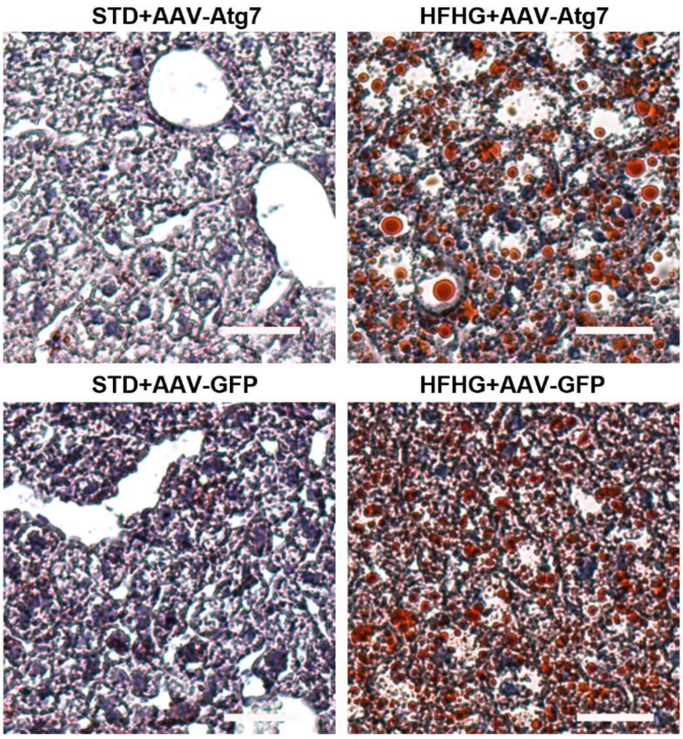

Supplemental Figure 7. Full-length gels of Western blot in Figure 1A and Figure 6A.

Figure 1A

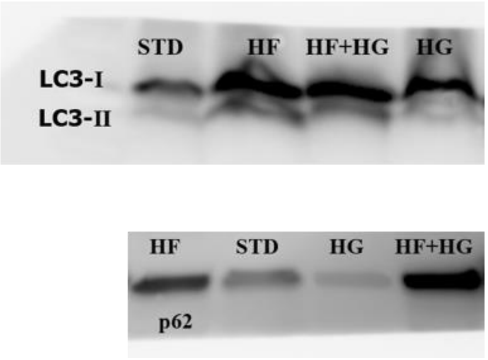

Figure 6A

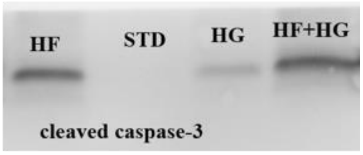

Supplement: Supplementary file 1 — Supplementary Information [file 41598_2017_16485_MOESM1_ESM.pdf]
